# Kott Gerontology Scholars Program Evaluation

**DOI:** 10.1093/geroni/igaf122.3801

**Published:** 2025-12-31

**Authors:** Heather Menne, Bailee Brekke, Jennifer Kinney, Lisa Peters-Beumer

**Affiliations:** Miami University, Oxford, Ohio, United States; Miami University, Waddington, New York, United States; Miami University, Oxford, Ohio, United States; Concordia University Chicago, River Forest, Illinois, United States

## Abstract

The Kott Gerontology Scholars Program (KGSP) has provided internship scholarships to promising advanced degree graduate students interested in the aging field who are attending Chicagoland area universities for 20 years. The KGSP encourages and fosters interdisciplinary cohorts of graduate students to develop hands-on skills that will engage and prepare them to continue working with and on behalf of older adults after graduation. The Scripps Gerontology Center conducted a mixed-methods approach to evaluate the overall program. Through the use of survey data (n = 74), 3 focus groups (n = 9), and key informant interviews (n = 5) with both alumni scholars and host agencies, the evaluation sought to a) assess the impact of KGSP on scholars’ career trajectories and professional development and b) evaluate the engagement and effectiveness of KGSP with partners’ agencies. It was evident that many KGSP scholars went on to pursue careers in the field, and 92% of Scholars working in the field of aging are likely or very likely to continue working in the field. Preliminary results from qualitative interviews suggest continued communication and networking opportunities for current and alumni scholars to engage in mentorship. Agency staff also recommended more communication and involvement with all of the current and previous agencies to support program growth. There is a recognized scarcity of gerontological expertise and there are significant gaps in the workforce in the field of aging. The KGSP helps prepare individuals to successfully enter and remain working in the aging network.

